# A case report of primary complex anal fistula with 7 external openings treated with combined preoperative 3D MRI model

**DOI:** 10.1097/MD.0000000000033264

**Published:** 2023-03-17

**Authors:** Yongping Hong, Yonggang Qiu, Guofeng Li

**Affiliations:** a Department of Anorectal Surgery, The First People’s Hospital of Xiaoshan District, Hangzhou, Zhejiang, P.R. China; b Department of Radiology Department, The First People’s Hospital of Xiaoshan District, Hangzhou, Zhejiang, P.R. China.

**Keywords:** 3-dimensional, fistula, imaging, magnetic resonance imaging

## Abstract

Anal fistula is a common perianal disease, but primary complex anal fistulas with 7 external openings is extremely rare.

**Patient concerns:**

We report a case of a 36-year-old man with a 10-year history of recurrent pus flow from paranal mass.

**Diagnosis:**

Primary complex anal fistula.

**Interventions:**

The patient underwent fistulotomy plus seton, which we successfully completed with the aid of 3-dimensional (3D) reconstruction model created from magnetic resonance imaging (MRI).

**Outcomes:**

The wound healed well and there was no recurrence 8 months after surgery.

**Conclusion:**

In the treatment of complex anal fistula, the combined application of 3D MRI model will be beneficial to obtain better surgical results.

## 1. Introduction

Complex anal fistulas were defined as trans-sphincteric fistulas involving > 30% of the external anal sphincter, suprasphincteric fistulas, horseshoe fistulas, anterolateral trans-perineal complex fistulas in female patients, and fistulas with combined inflammatory bowel disease, radiation enteritis, malignant tumors, anal incompetence, chronic diarrhea.^[1–4]^ Complex anal fistulas with multiple external openings (>7 external openings) are rare and only 1 case report present a secondary tuberculous anal fistula with 8 external openings.^[5]^ We report here a case of primary 7 external openings complex anal fistula with complete resection assisted by preoperative 3-dimensional (3D) magnetic resonance imaging (MRI) model. This case report presents a new treatment strategy for complex anal fistulas.

## 2. Case presentation

**Figure 1.**
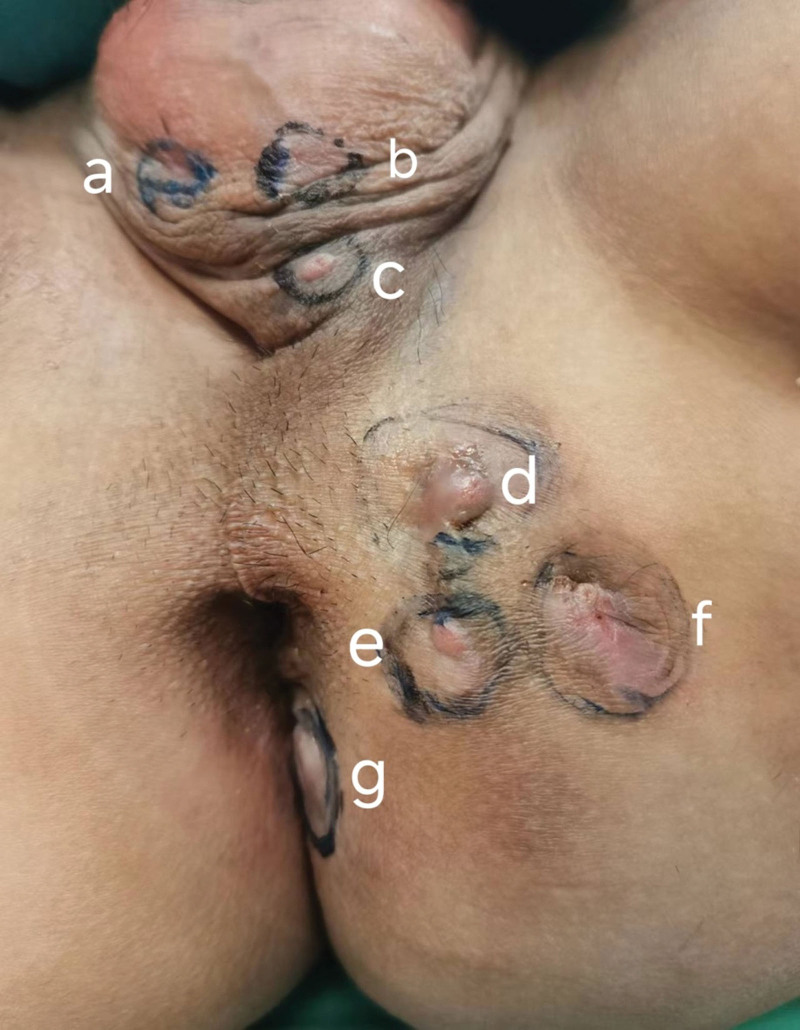
Preoperative patient’s complex anal fistula external orifice markings. (a–c) Scrotal lateral external openings; (d–f) 3 o’clock site’s external openings; (g) 5 o’clock site’s external opening.

The patient, a 36-year-old male, was admitted to the hospital with a perianal mass with recurrent pus flow for more than 10 years. Over the past 10 years, the perianal masses occasionally increased in size and added multiple perianal masses with pain and ruptured pus, during which the patient received antiinflammatory treatment, but with poor results. Physical examination: the patient was placed in a lithotomy position, and a total of 7 masses were visible at 12, 1 to 3, and 5 o’clock sites, about 2 to 7 cm from the anus, with light pressure pain, ulcerated and flowing pus, and a cord-like mass extending into the corresponding anus (Fig. 1). No other masses were palpated by rectal palpation. Laboratory test and colonoscopy results were not abnormal. The patient denied a history of tuberculosis and secretion culture negative for mycobacterium tuberculosis. Enhanced MRI of the anal canal showed that 2 canal-like structures were seen on the left side of the anal canal, with low T1 and high T2 signal, with restricted diffusion and significant enhancement, and the internal opening was about 2 o’clock and 7 o’clock sites, one of which traveled posteriorly along the sphincter gap, and the other traveled forward through the external anal sphincter. Because the preoperative colonoscopy was unremarkable and inflammatory bowel disease was ruled out, the diagnosis of primary complex anal fistula was made.

**Figure 2.**
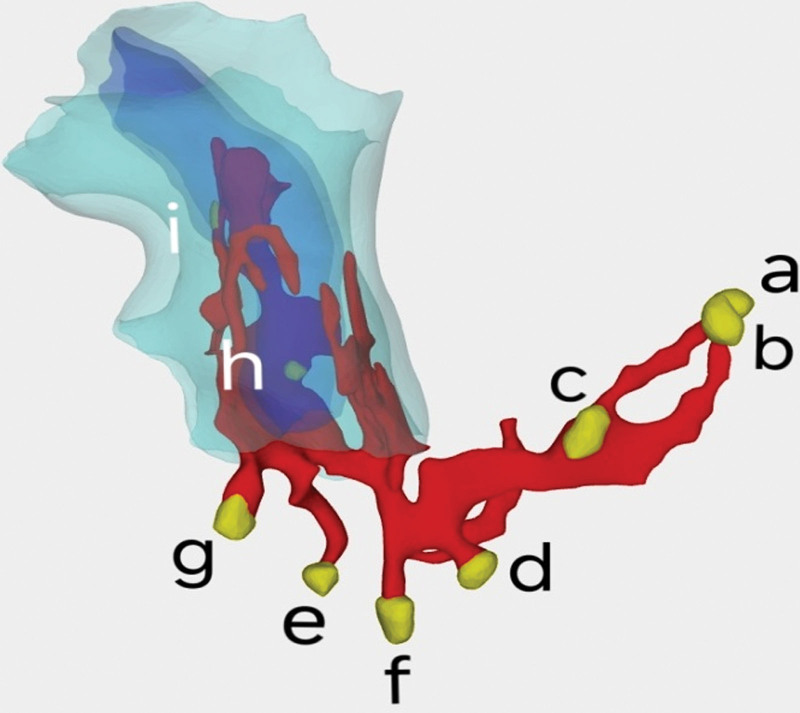
Preoperative 3D reconstruction of the patient with complex anal fistula. (a–c) Scrotal lateral external openings; (d–f) 3 o’clock site’s external openings; (g) 5 o’clock site’s external opening; (h) 1 o’clock site’s internal opening; (i) 7 o’clock site’s internal opening; Dark blue: internal sphincter; light blue: external sphincter.

We exported the MRI images and used the software for 3D reconstruction to show (Fig. 2) a branching fistula in the skin at 5 o’clock site about 3 cm from the anal opening in the lithotomy position, one extending deeper along the inner and outer sphincter and the internal opening at 7 o’clock site above the anal sphincter in the intestinal mucosa. The other fistula extends toward 2 o’clock site and divides into 2 fistulas at this location, one extending deeper through the internal sphincter opening at the 2 o’clock site dentition. The other extends left through the external sphincter to 3 to 4 o’clock sites and divides into 4 branches, 3 of which open 3 to 5 cm lateral to the skin at 1 to 3 o’clock sites, respectively. The other branch extends in the direction of the anterior scrotum and divides into a total of 3 branches at the root of the scrotum, each opening at the scrotum.

**Figure 3.**
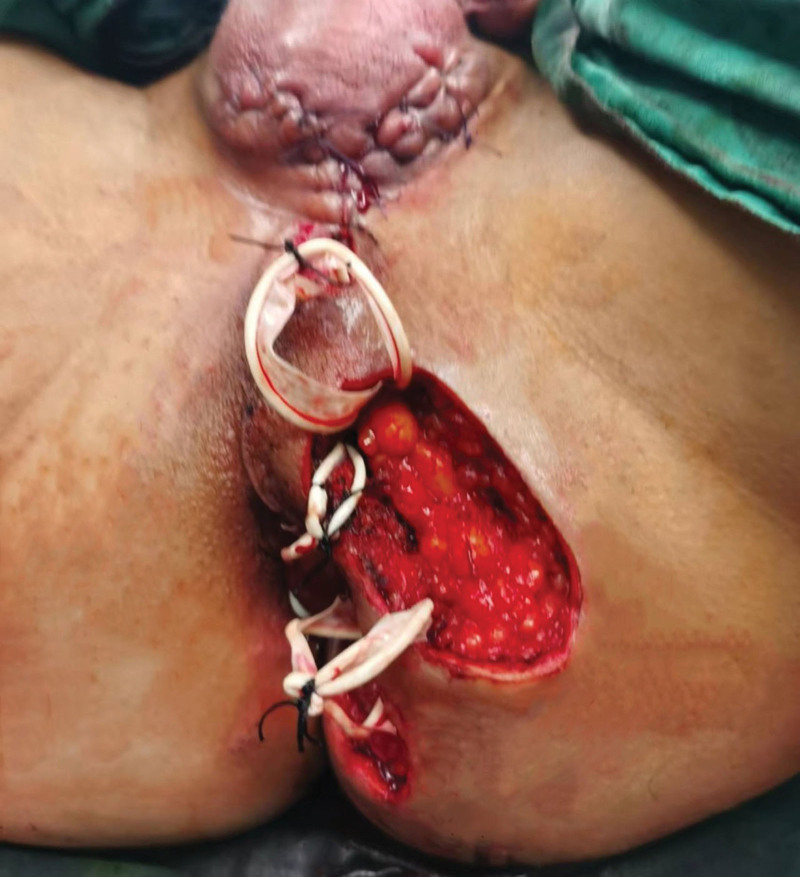
Wound condition after completion of surgery.

**Figure 4.**
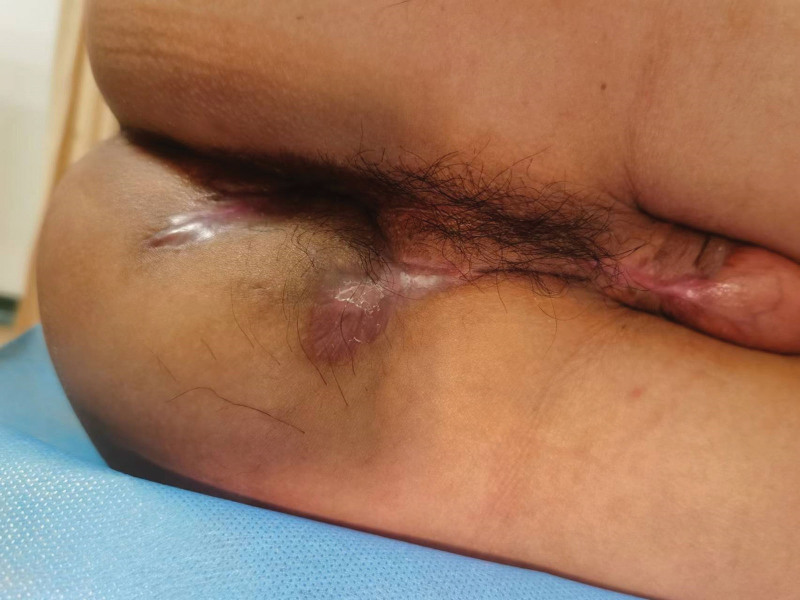
Wound healing of the patient 4 months after surgery.

The patient then underwent fistulotomy plus seton (Fig. 3), which we successfully completed preoperatively and intraoperatively with the aid of 3D reconstruction model. The threads at 2 o’clock and 5 o’clock sites through the internal opening served as drainage and slow cutting, while the other threads only served as drainage. At 8 weeks postoperatively, all threads had completely fallen off, and at 10 weeks postoperatively, the patient’s wounds had completely healed without significant discomfort. The patient was followed up until 8 months after surgery, no recurrence was observed and the wound healed well (Fig. 4).

## 3. Discussion

The treatment of complex anal fistulas is a major challenge for surgeons because the complexity of fistulae travel can lead to difficult surgery, a high rate of postoperative recurrence, and a tendency to impair anal function. If anal incontinence occurs, it will seriously affect the quality of life of the patient, who may even suffer from depression and social isolation. There are 3 basic principles for the surgical treatment of anal fistulae, including accurate identification of the fistula and fistulae internal opening, removal of the fistula and preservation of anal sphincter function. Therefore, preoperative diagnosis and anatomic identification of the fistula is particularly important. Therefore, preoperative diagnosis and anatomic identification of anal fistulas are particularly important. At present, with the popularization of intracavitary ultrasound and MRI, imaging can provide a more objective diagnostic basis for the diagnosis of anal fistula and improve the accuracy of the diagnosis of anal fistula. At this stage, MRI has become the gold standard for the diagnosis of anal fistula.^[6]^ MRI can clearly show the location of the internal opening of the fistula and the direction of fistula travel, and accurately classify the fistula, thus providing a reliable basis for further clinical treatment. The process of 3D reconstruction is to export the 2D image of MRI, then the radiologist will depict the fistula location, and finally import the software for 3D reconstruction to obtain a 3D image that can clearly show the anatomical structure of the lesion, which is important for intraoperative guidance. This is a rare case of primary 7-external openings complex anal fistula with a 10-year duration and an extremely complex fistula pathway. The procedure is extremely difficult and requires a high degree of preoperative anatomical familiarity. If the location of the fistula is incorrectly estimated, not only will the main lesion remain, leading to postoperative recurrence, but there is also the possibility of irreversible damage to the anal sphincter caused by the enlarged incision and even the occurrence of anal incontinence. The preoperative 3D visualization of the fistula gives us a clearer visualization of fistula anatomy in relation to the sphincter complex, and helps us to choose the most appropriate type of surgery for the patient, reducing tissue damage and bleeding, while allowing us to shorten the operation time. At the same time, the lesion was compared with the 3D image in real time to precisely guide the operation, especially in determining the height of the 7 o’clock site’s internal opening, the angle of its fistula, and the anatomical position between the fistula and the sphincter at 2 o’clock site, which led to the successful completion of the operation.

Currently, there are surgical methods for treating anal fistulas, including those that damage the sphincter such as: anal fistulotomy, fistulectomy, and seton, while the main surgical approaches to protect the function of the sphincter are: ligation of the intersphincteric fistula tract was first proposed by Rojanasakul^[7]^ in 2007 to ligate and disconnect the fistula through a special anatomical area, the inter-sphincter sulcus, and to remove the distal fistula, which can this procedure minimizes the damage to the sphincter muscle fibers. Video-assisted anal fistula treatment uses a thin-lumen fistuloscope to enter the fistula through the external opening, displaying the anatomy of the fistula clearly and dynamically on the screen, destroying the inner wall of the fistula with electrocautery, and finally closing the internal opening and draining the infected tissue from the lumen. The main advantages of video-assisted anal fistula treatment are small invasion, direct visualization, and avoidance of sphincter injury.^[8]^ The anal fistula plug is a protein mixture, the main component of which is derived from decidual cells of the submucosa of the porcine small intestine. Its insertion into the fistula can both close the internal opening and provide a scaffold for fibroblast growth to promote wound repair and healing. Early reports showed good efficacy (70%–100% cure rate).^[9–11]^ However, later studies and their improved methods failed to replicate these results. Multicenter studies and Meta-analysis suggest that the success rate is <50%.^[12,13]^ There are also surgeries for sphincter protection such as anorectal advancement flap, laser closure of anal fistula and so on.

Therefore, in the treatment of complex anal fistula, it is necessary not only to choose the appropriate surgical method, but also to strengthen multidisciplinary communication and cooperation. The preoperative use of 3D reconstruction technology in imaging can guide surgeons to operate more precisely, reducing tissue damage and bleeding and shortening the operating time, thus increasing the cure rate of complex anal fistula and reducing recurrence. This primary fistula has a long course, evolving over a period of 10 years into a complex fistula with 7 external openings, which reminds us that fistulas should be treated early to avoid aggravation of the condition.

## Author contributions

**Data curation:** Yonggang Qiu.

**Visualization:** Yonggang Qiu.

**Writing – original draft:** Yongping Hong.

**Writing – review & editing:** Guofeng Li.
